# Ethylene response pathway modulates attractiveness of plant roots to soybean cyst nematode *Heterodera glycines*

**DOI:** 10.1038/srep41282

**Published:** 2017-01-23

**Authors:** Yanfeng Hu, Jia You, Chunjie Li, Valerie M. Williamson, Congli Wang

**Affiliations:** 1 Key Laboratory of Mollisols Agroecology, Northeast Institute of Geography and Agroecology, Chinese Academy of Sciences, Harbin 150081 China; 2 Department of Plant Pathology, University of California, Davis, CA 95616 USA

## Abstract

Plant parasitic nematodes respond to root exudates to locate their host roots. In our studies second stage juveniles of *Heterodera glycines*, the soybean cyst nematode (SCN), quickly migrated to soybean roots in Pluronic F-127 gel. Roots of soybean and non-host *Arabidopsis* treated with the ethylene (ET)-synthesis inhibitor aminoethoxyvinylglycine (AVG) were more attractive to SCN than untreated roots, and significantly more nematodes penetrated into roots. Moreover, *Arabidopsis* ET insensitive mutants (*ein2, ein2-1, ein2-5, ein3-1, ein5-1*, and *ein6*) were more attractive than wild-type plants. Conversely, the constitutive triple-response mutant *ctr1-1*, was less attractive to SCN. While ET receptor gain-of-function mutant *ein4-1* attracted more SCN than the wild-type, there were no significant differences in attractiveness between another gain-of-function ET receptor mutant, *etr1-3*, or the loss-of-function mutants *etr1-7* and *ers1-3* and the wild type. Expression of the reporter construct EBS: β-glucuronidase (*GUS*) was detected in *Arabidopsis* root tips as early as 6 h post infection, indicating that ET signaling was activated in *Arabidopsis* early by SCN infection. These results suggest that an active ET signaling pathway reduces root attractiveness to SCN in a way similar to that reported for root-knot nematodes, but opposite to that suggested for the sugar beet cyst nematode *Heterodera schachtii*.

Soybean (*Glycine max*) is an important food crop and provides a sustainable source of protein and oil worldwide. The soybean cyst nematode (SCN, *Heterodera glycines*), a sedentary root parasite, is one of the most economically important pathogens of soybean and causes a devastating disease in this crop around the world^1^,^2^. Once SCN second stage juveniles (J2) hatch from eggs, they must locate host roots and start feeding before their energy reserves are depleted^3^. However, the signals attracting J2 to the host root remain unclear. After the infective J2 reach host roots, they penetrate into the root and migrate intracellularly through the cortical layer to the vascular cylinder utilizing cell wall-degrading enzymes released through their stylets. The J2 then select individual cells in the pericycle region to initiate formation of specialized feeding sites called syncytia, which provide nutrients for the growth and development of the sedentary nematode^4^,^5^.

J2 recognize chemical signals in the soil environment by using their amphids, the nematode’s main chemoreception organ^6^,^7^. Behavioral studies have shown that plant parasitic nematodes are attracted to host root secretions^8^,^9^,^10^. Some specific compounds in root exudates such as tannic acid, flavonoids, glycosides, and fatty acids that may contribute to J2 repulsion or attraction have been identified^11^,^12^,^13^. Lauric acid from crown daisy root exudate appears to mediate chemotaxis of the root-knot nematode (RKN) *Meloidogyne incognita* through a peptide neuromodulator encoded by the gene *Mi-flp-18*. Interestingly, this compound is an attractant at low concentrations but a repellant at high concentrations^14^. There is also evidence that attractants and repellents are produced by other rhizosphere organisms, including J2 themselves^15^,^16^,^17^,^18^. In addition, gradients of chemical and physical factors such as CO_2_, pH, ions, cyanide, and temperature in the rhizosphere have been reported to contribute to attraction or repellent activity of plant nematodes^19^,^20^,^21^.

Plant hormones not only regulate plant growth and development but also play a key role in plant responses to environmental stimuli including biotic and abiotic stresses. Transcriptome and microarray analyses of host roots infected with plant nematodes showed differential changes in the expression of genes responding to several hormones including auxin, ethylene (ET), jasmonic acid (JA), salicylic acid (SA), brassinosteroids (BR), gibberellins (GA), and abscisic acid (ABA)^22^,^23^,^24^. A number of studies have indicated that the signaling pathways of SA, JA, BR, ABA, and ET are involved in the defense response of host plants to nematodes^25^,^26^,^27^,^28^,^29^,^30^. Auxin, ET and GA have also been reported to play a positive role in the early stages of development of nematode feeding cells^31^,^32^,^33^. ET is recognized to play a role in nematode attraction, migration, feeding site formation, and host defense during early stages of nematode infection^32^,^34^,^35^,^36^, but the role may differ for the interaction of plants with cyst nematode and RKN. For example, an intact ET pathway is prerequisite for plant defense to RKN^27^,^37^. In contrast, the ET pathway facilitates cyst nematode parasitism as evidenced by the significant reduction of cyst nematodes inside the roots of ET-insensitive mutants or wild type plants treated with ET inhibitors^31^,^36^. ET is also known to play an important role in *H. glycines* development^38^,^39^.

Over the past few decades, the ET signal transduction pathway has been extensively investigated in the model plant *Arabidopsis*. Ethylene is perceived by five membrane receptors ETHYLENE RESPONSE1 (ETR1), ETHYLENE RESPONSE SENSOR1 (ERS1), ETR2, ERS2, and ETHYLENE INSENSITIVE4 (EIN4), which are negative regulators of ET responses; ET binding to these receptors represses the negative regulation^40^,^41^,^42^,^43^. In the absence of ET, the active receptors recruit another negative regulator of ET signaling, Constitutive Triple Response1 (CTR1), to phosphorylate the C-terminal domain of EIN2. and promote the binding of EIN2 to the F-box proteins EIN2 TARGETING PROTEINS, ETP1 and ETP2. This interaction leads to the ubiquitylation and degradation of EIN2 by 26S proteasome, which subsequently represses the EIN3/ETHYLENE-INSENSITIVE3-LIKE (EIL)-dependent transcriptional cascade^44^,^45^,^46^,^47^. EIN2 is a central positive regulator of ethylene responses, and its null mutant *ein2* is completely insensitive to ET^48^. EIN2 can directly activate the EIN3/EIL1 transcription factors, which results in EIN3/EIL1 binding to the promoters of ET response genes ERF1 or other downstream genes to activate or repress their expression^49^,^50^, thereby regulating ET responses in plants.

While several studies have focused on the role of hormones in the later stages of nematode infection, only a few studies have investigated their contribution to mediating nematode attraction and host-seeking behaviors. Using *Arabidopsis* mutant lines in ET signaling and perception, one study concluded that active ET signaling played a positive role in the attraction of the sugar beet cyst nematode (SBCN) *Heterodera schachtii* to *Arabidoposis*^32^. However, in another study, ethylene signaling was found to negatively affect attraction of the RKN, *M. hapla*^35^.

In the present study, we investigated the role of the ET signaling pathway in the attraction of *H. glycines* to soybean and the non-host plant *Arabidopsis* by using a Pluronic F-127 (PF-127) gel attraction assay^20^,^35^. We found that soybean and *Arabidopsis* roots treated with the ET-synthesis inhibitor aminoethoxyvinylglycine (AVG) were more attractive to J2 than untreated roots. Our studies of *Arabidopsis* mutants in ET perception and signaling further suggested that an active signaling pathway reduces attractiveness of plant roots to SCN in a similar way as to RKN, but opposite to the results reported for *H. schachtii*.

## Results

### Attraction of *Heterodera glycines* to soybean roots

**Figure 1:**
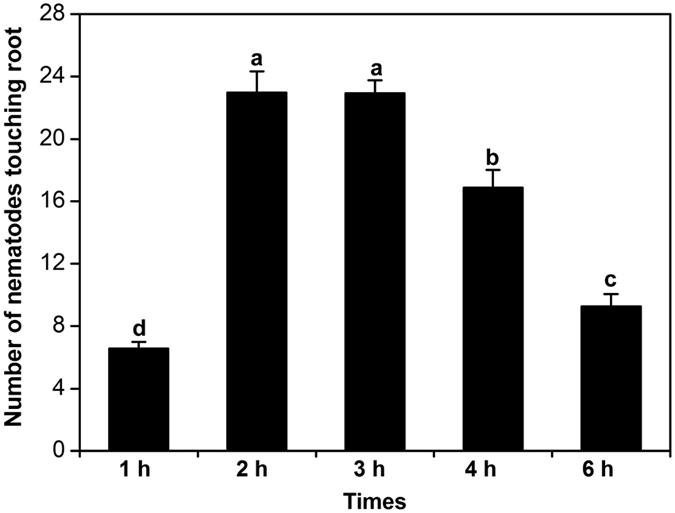
Attraction of *Heterodera glycines* to soybean root tips. The number of J2 touching the terminal 5 mm of the roots of soybean seedlings was counted at 1, 2, 3, 4, and 6 h after placing root tips in 2 ml Pluronic F-127 gel containing 200 J2. Bars represent the mean ± SE of one representative experiment (n = 24). Bars with different letters indicate significant differences *(P* < *0.0*5, Tukey’s *t* Test). The experiments were repeated three times with similar results.

Second-stage juveniles of *H. glycines* were observed to move toward soybean roots within 1 h post exposure in the PF-127 gel attraction assay. Nematode J2 close to root tips moved to the root surface and started to penetrate into the roots. The numbers of J2 touching the root tips of soybean cv. Dongsheng 1 at 2 h and 3 h post exposure were significantly greater than those at 1 h, 4 h, and 6 h post exposure (Fig. 1). The decline in numbers at the latter time points was due to nematode penetration into the roots. Therefore, we selected the 2-h time point for the attraction assay in the subsequent experiments.

**Figure 2:**
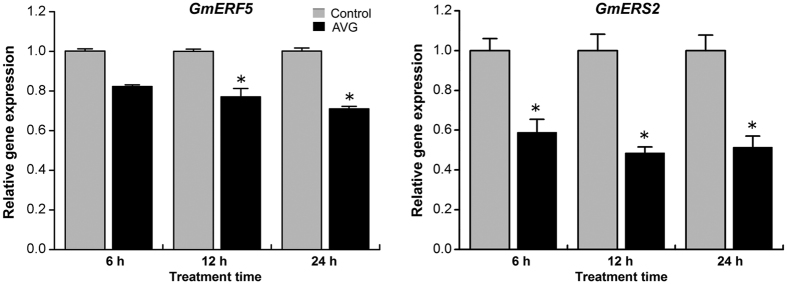
Quantitative RT-PCR analysis of the effects of AVG on ethylene-responsive gene expression in soybean roots. RNA was extracted from roots at 6, 12, and 24 h after treatment with AVG and from control roots. Gene expression levels were normalized using the internal reference gene *GmUBQ3*. Data are mean ± SE of three independent experiments, asterisks indicate significant different expression levels in comparison with the control roots (*P < *0.05*).

We proposed to treat soybean roots with the ET-synthesis inhibitor AVG prior to the attraction assay as a strategy to evaluate whether ET signaling played a role in attractiveness of soybean roots to *H. glycines*. To confirm that AVG treatment of soybean roots under our conditions had the anticipated effect, the expression of ethylene-response marker genes *GmERF5* and *GmERS2* was measured by qRT-PCR after 6, 12 and 24 h of treatment with AVG. *GmERF5* transcript levels were significantly reduced compared to untreated roots at 12 and 24 h after AVG treatment, whereas *GmERS2* levels were reduced at all three time points (Fig. 2). These results indicated the AVG-treatment down-regulated expression of the ethylene-response pathway in soybean roots.

**Figure 3:**
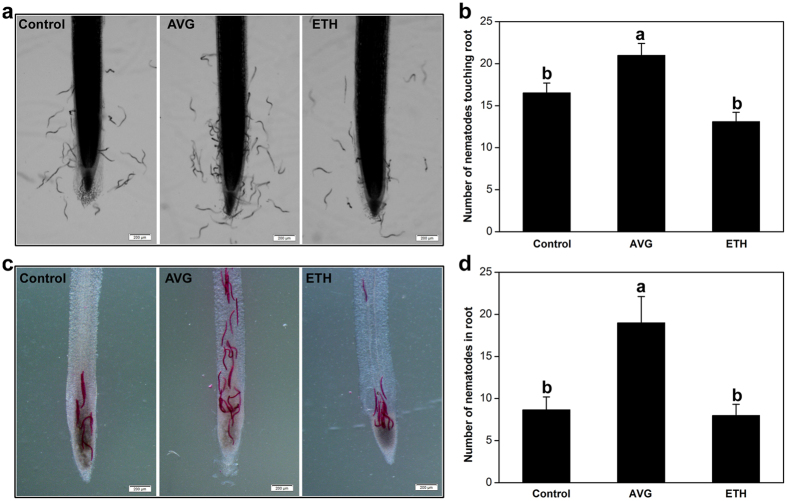
Effects of AVG and ethephon treatments on the attractiveness of soybean root to *Heterodera glycines.* The roots of 12-day-old soybean seedlings pretreated with water (Control), 50 μM AVG, or 200 μM ETH for 24 h were excised and placed into Pluronic F-127 gel containing 200 J2. (**a**) Microscopic observation of nematodes and root tips at 2 h after assay start. (**b**) The number of nematodes touching the root within the terminal 5 mm at 2 h after assay start. (**c**) Soybean roots stained with acid fuchsin, which stains nematodes red, at 6 h after assay start. (**d**) The number of stained nematodes inside the root at 6 h after assay start. Bars are the mean ± SE from one representative experiment (n = 24). The experiments were repeated three times with similar results. Bars with different letters indicate significant differences *(P* < *0.0*5, Tukey’s *t* Test). Scale bar = 200 μm.

More nematodes were attracted to soybean root tips that had been pretreated with AVG for 24 h than to untreated roots (Fig. 3a). To exclude the possibility that the increased attractiveness of AVG-treated roots was the result of nematode chemotaxis to AVG, we tested the response of J2 to AVG and found that there was no difference in nematode chemotaxis toward AVG compared to a water control (data not shown). We also treated soybean roots with the ET analog ethephon (ETH)^27^,^36^,^51^, but no significant difference was seen between the number of nematodes touching the ETH-treated and water-treated root tips (Fig. 3a,b). The number of nematodes inside root tips treated with AVG or ETH was counted at 6 h after assay start and following staining with acid fuchsin. Exogenous application of AVG resulted in a significant increase in nematode numbers inside soybean root, while no change was found following ETH treatment (Fig. 3c,d).

### Attraction of *Heterodera glycines* to Arabidopsis roots

**Figure 4:**
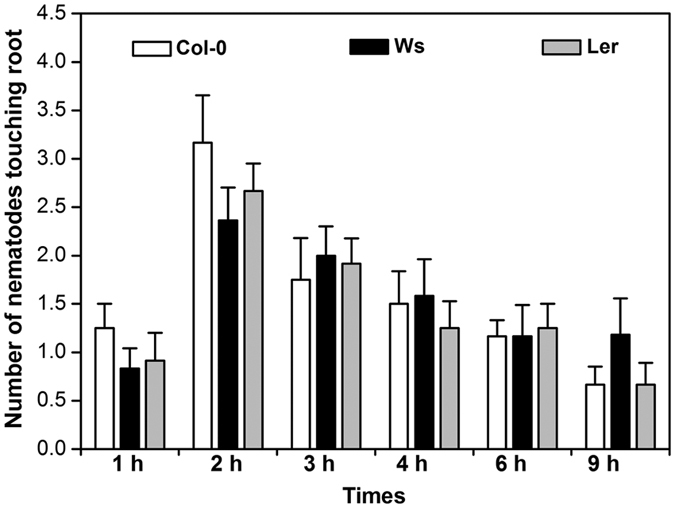
Attraction of *Heterodera glycines* to *Arabidopsis* root tips. The number of J2 nematodes touching the terminal 5 mm of the root tips of wild-type *Arabidopsis* (Col-0, Ws and Ler) was counted at 1, 2, 3, 4, 6 and 9 h after assay start in Pluronic F-127 gel. Values are the mean ± SE of one representative experiment (n = 24). The experiments were repeated three times with similar results.

We tested whether *H. glycines* was attracted to roots of the non-host plant *Arabidopsis*. The number of SCN J2 touching root tips of *Arabidopsis* ecotypes Col-0, Ler and Ws was counted at 1, 2, 3, 4, 6, and 9 h post exposure. The greatest (*P* < *0.05*) number of nematodes touching the root of Col-0 was detectable at 2 h (Fig. 4). At 9 h post exposure, the number of nematodes touching the root declined to the level at 1 h post exposure. The three *Arabidopsis* ecotypes tested showed a similar pattern of attractiveness to SCN J2. This result indicates that the roots of *Arabidopsis* also secrete chemicals that attract *H. glycines*. Based on these results, the 2-h time point was chosen to evaluate attraction in subsequent experiments.

**Figure 5:**
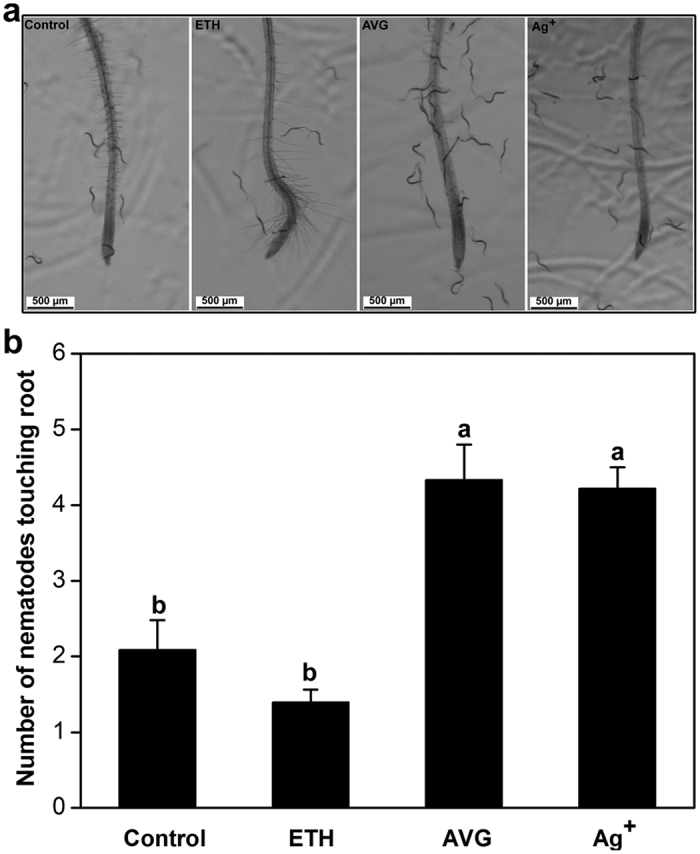
Effects of chemical modulation of ethylene levels or signaling on the attractiveness of *Arabidopsis* roots to *Heterodera glycines*. The roots of 7-day-old *Arabidopsis* seedlings (Col-0) pretreated with water (Control), 200 μM ethephon (ETH), 10 μM AgNO_3_ or 50 μM AVG for 24 h were excised and placed into Pluronic F-127 gel containing 300 J2. Nematodes and root tips of *Arabidopsis* at 2 h after assay start (**a**). The number of nematodes touching the root within the terminal 5 mm was counted (**b**) at 2 h after assay start. Bars are the mean ± SE from one representative experiment (n = 24). These experiments were repeated three times with similar results. Bars with different letters indicate significant differences (*P* < *0.05*, Tukey’s *t* Test). Scale bar = 500 μm.

Similar to our results with soybean, AVG-treated *Arabidopsis* roots showed a significantly higher attractiveness to SCN J2 than the control roots, and no differences were found in the attractiveness of ETH-treated roots and non-treated roots (Fig. 5a,b). As another test of the effect of ET action on *Arabidopsis* root attractiveness, we treated seedlings with AgNO_3_ (Ag^+^), which inhibits ET perception by inactivating ET receptors^52^,^53^. Ag^+^-treated roots were observed to attract more nematodes than control roots (Fig. 5a,b).

### Attractiveness of *Arabidopsis* ET mutants to *Heterodera glycines*

**Figure 6:**
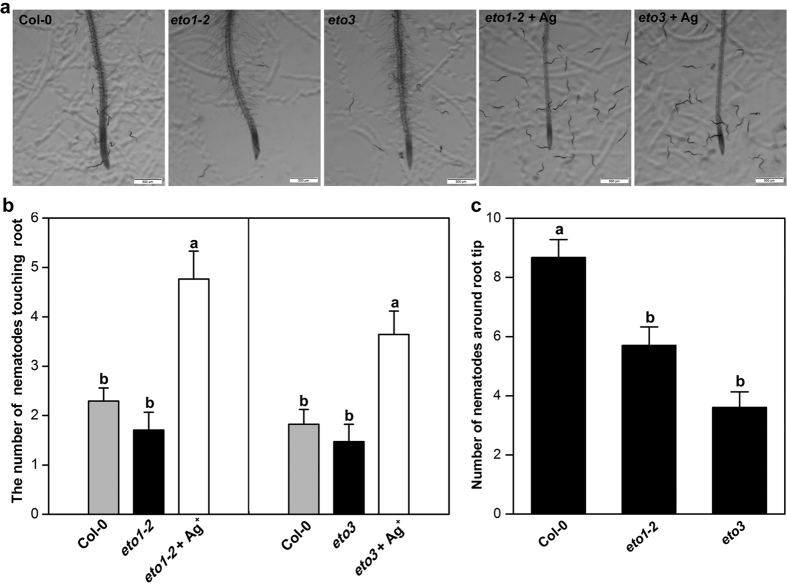
Response of *Heterodera glycines* to *Arabidopsis* ethylene-overproducing mutants. (**a**) Representative images showing the response of nematodes to the roots of ethylene-overproducing *Arabidopsis* mutants with or without Ag^+^ at 2 h after placing seedlings in Pluronic F-127 gel containing 300 J2. Scale bar = 500 μm. (**b**) The number of nematodes touching the terminal 5 mm of the root at 2 h after starting the assay. (**c**) The number of nematodes within a circle of 5 mm diameter centered at 2.5 mm proximal to the root tip was counted at 2 h after starting the assay. Bars represent mean ± SE of one representative experiment (n = 24). Bars with different letters are statistically different using Tukey’s *t* Test (*P* < *0.05*).

Because chemical modulation of ET in *Arabidopsis* and in soybean affects SCN attraction in a similar way, we tested *Arabidopsis* mutants in ET biosynthesis, perception, and downstream signal transduction. Root tips of *Arabidopsis* mutants *eto1-2* and *eto3*, which produce more ethylene than WT^44^,^54^, did not show a significant difference in the number of nematodes touching the roots compared to WT (Fig. 6a,b). However, it appeared that fewer nematodes were found near the roots of *eto1-2* and *eto3* than of WT (Fig. 6a). To test this further, we counted J2 within a circle of 5 mm diameter centered at 2.5 mm above the root tip and found that, indeed, the numbers of J2 near the *eto1-2* and *eto3* root tips were lower than that in WT (Fig. 6c). This may suggest that a signal produced at a different level in the mutant plants is perceived at a distance from the roots. The phenotypes of shorter roots and more and longer root hair characteristic of *eto1-2* and *eto3* are known to be restored to the wild-type phenotype by treatment with Ag^+ ^55 and we found that Ag-treated *eto1-2* and *eto3* mutants become more attractive to J2 than non-treated mutant or even wild type plants (Fig. 6a,b). This supports a role of ET-receptor mediated signaling in reducing attraction of the root tip to J2.

**Figure 7:**
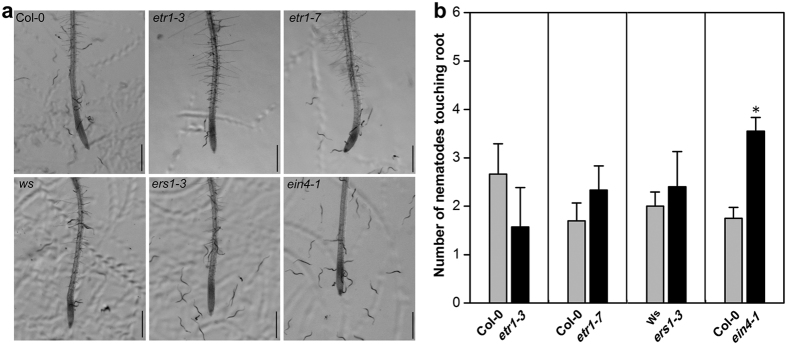
Attraction of *Heterodera glycines* to root tips of *Arabidopsis* ethylene receptor mutants. Response of J2 was compared to roots of WT, two GOF ET receptor mutants (*etr1-1* and *ein4-1*), and two LOF ET receptor mutants (*etr1-7* and *ers1-3*). (**a**) Images were taken 2 h after inoculation of seedlings in 1 ml of Pluronic F-127 gel containing 300 J2. Scale bar = 200 μm. (**b**) Number of nematodes touching the terminal 5 mm of the root was counted at 2 h after placing roots in Pluronic F-127 gel containing 300 J2. Bars represent mean ± SE of one representative experiment (n = 24). Asterisks represent statistically significant differences with wild-type *Arabidopsis* (Col-0 or Ws) using Student’s t-test (*P < *0.05*). The experiments were repeated three times with similar results.

ET sensing in *Arabidopsis* occurs through a family of ET receptor proteins, and dominant gain-of-function mutations in the encoding genes that confer ethylene insensitivity are available^40^,^41^,^43^. We tested two gain-of-function mutants for effect on SCN J2 attraction, *etr1-3* did not differ in attractiveness from WT, while *ein4-1* was more attractive. Furthermore, loss-of-function ET receptor mutants *etr1-7* and *ers1-3,* which have enhanced ET sensitivity, did not exhibit obvious differences in attractiveness from WT (Fig. 7a,b).

**Figure 8:**
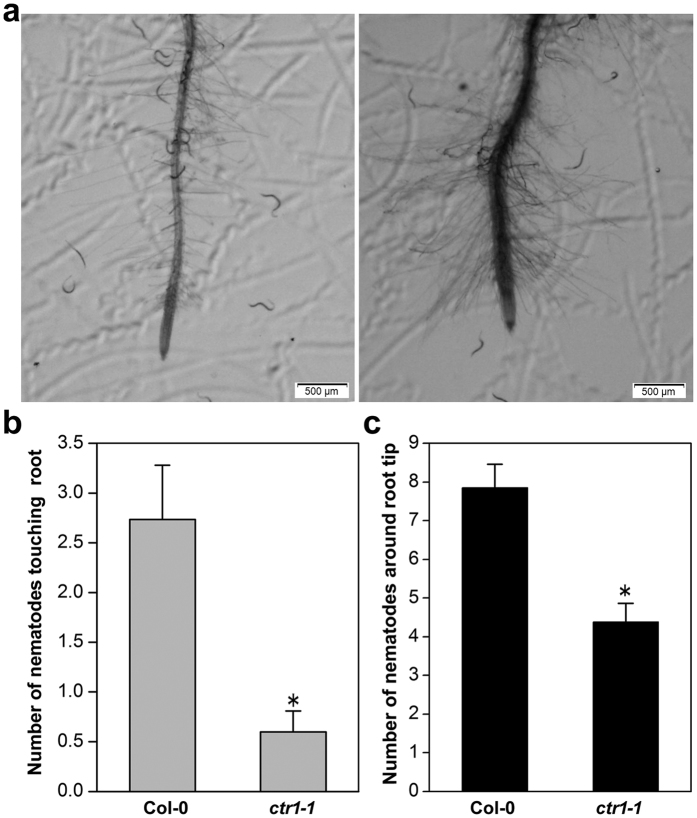
Response of *Heterodera glycines* to the constitutive ET-response mutant *ctr1-1*. (**a**) Representative images showing the response of nematodes to the roots of *ctr1-1* in Pluronic F-127 gel containing 300 J2. Scale bar = 500 μm (**b**) The number of nematodes touching the terminal 5 mm of the root was counted at 2 h after starting the assay. (**c**) The number of nematodes within a circle of 5 mm diameter centered at 2.5 mm above the root tip at 2 h after starting the assay. Bars represent mean ± SE of one representative experiment (n = 24). Asterisks represent statistically significant differences from wild-type *Arabidopsis* using Student’s t-test (*P < *0.05*).

**Figure 9:**
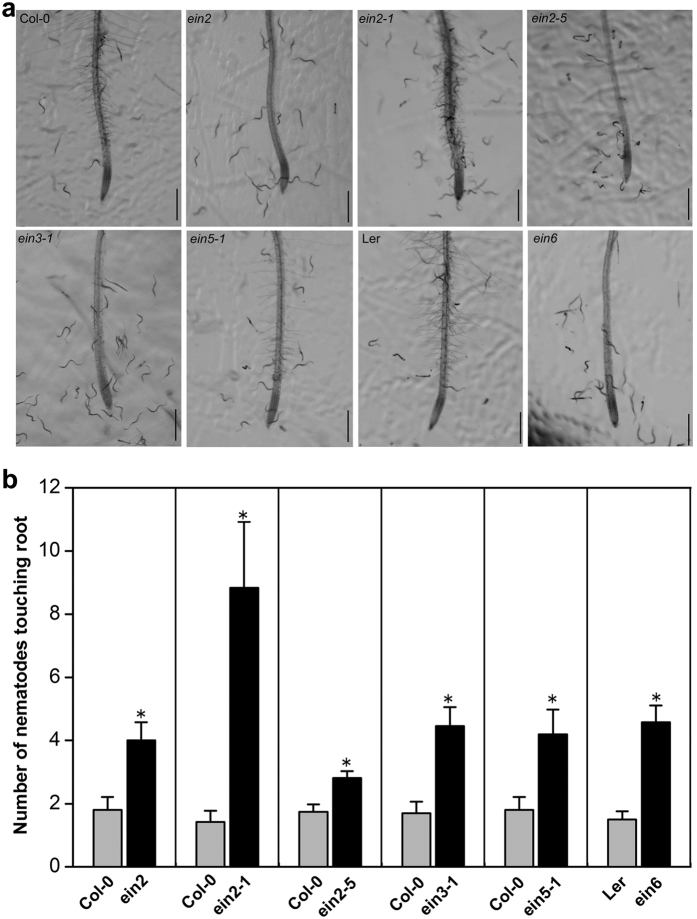
Attraction of *Heterodera glycines* to the root tips of ethylene-insensitive *Arabidopsis* mutants. (**a**) Representative images showing the response of nematodes to *Arabidopsis* mutants defective in positive regulation of the ET signaling pathway. Images were taken 2 h after placing seedlings in 1 ml of Pluronic F-127 gel containing 300 J2. Scale bar = 200 μm. (**b**) The number of nematodes touching the terminal 5 mm of the root was counted at 2 h after assay start. Bars represent mean ± SE of one representative experiment (n = 24). Asterisks represent significant differences compared to wild-type *Arabidopsis* (Col-0 or Ler) using Student’s t-test (*P < *0.05*). The experiments were repeated three times with similar results.

CTR1 is a negative regulator of the ET response, and the loss-of-function mutant *ctr1-1* has the constitutive triple-response phenotype^44^. Interestingly, *ctr1-1* attracted significantly fewer J2 than WT (Fig. 8), and the number of nematodes touching roots or in the vicinity of the root tip was significantly lower (*P* < *0.05*) than that in WT (Fig. 8b,c). Further, ET-insensitive mutants *ein2, ein2-1, ein2-5, ein3-1, ein5-1*, and *ein6*, which are defective in positive regulation of ET signaling, showed significantly higher attractiveness compared with wild-type plants (Fig. 9a,b).

### *Heterodera glycines* infection activates ET signaling

**Figure 10:**
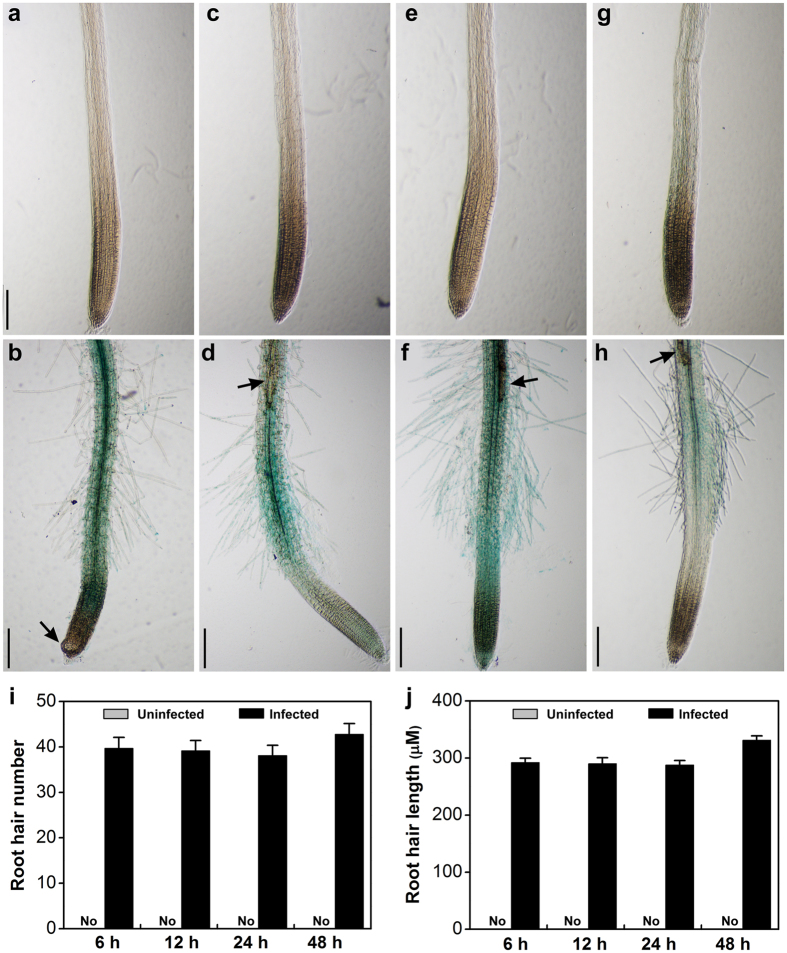
Activation of ethylene signaling in *Arabidopsis* roots upon *Heterodera glycines* infection. Expression of GUS in *EBS::GUS Arabidopsis* roots in uninfected or nematode-infected roots at 6 (**a**,**b**), 12 (**c**,**d**), 24 (**e**,**f**), and 48 h (**g**,**h**) after inoculation. Scale bar = 100 μm. Arrowheads point to the nematode. Root hair numbers (**i**) and root hair length (**j**) for the 500-μm zone of the root tip were counted from 10 seedlings. ‘No’ indicates no root hairs were seen. The data represent the mean ± SE of one representative experiment. The experiments were repeated three times with similar results.

Previous studies indicated that infective J2 of *H. glycines* could penetrate and migrate inside the *Arabidopsis* roots in the same manner as *H. schachtii*^56^. To assess whether ET signaling is activated in *Arabidopsis* early in the response to *H. glycines* infection, we examined the expression levels in transgenic *Arabidopsis* carrying the ET reporter construct, *EBS::GUS*, in which the β-glucuronidase (*GUS*) reporter gene is driven by a synthetic *EIN3*-responsive promoter^57^. No visible expression of *EBS::GUS* was observed in uninfected roots at tested time points (Fig. 10a,c,e and g). At 6 h post inoculation with *H. glycines*, we observed that J2 had penetrated and migrated inside the root and that there was a marked increase in *GUS* expression in the majority of root infection sites (Fig. 10b). Strong *GUS* expression was also observed in the infection sites and surrounding cells at 12 and 24 h after infection (Fig. 10d,f). However, the nematode-induced increase in *GUS* expression in the infection sites was reduced at 48 h post infection (Fig. 10h). We also noted a dramatic increase in the number and length of root hairs for infected *EBS::GUS* seedlings compared to uninfected roots (Fig. 10). ET plays a vital role in root hair formation and elongation^58^, and this increase further supports activation of ET signaling early during the infection process.

## Discussion

In this study, the increased attractiveness of SCN to the roots of the host soybean and non-host *Arabidopsis* in the presence of the ET biosynthesis inhibitor AVG indicated that ET levels or signaling modulated the root attractiveness to SCN. We explored available *Arabidopsis* mutants in ET synthesis and signaling to gain additional information on whether ET levels or signaling were responsible. *Arabidopsis* mutants that had reduced ET signaling (*ein4-1, ein2, ein2-1, ein2-5, ein3-1, ein5, ein6*) were more attractive compared to wild type, indicating that reduced ET signaling in the plant played a positive role in the attraction of SCN to roots. Of the two GOF ET-receptor mutants tested, *ein4-1* was significantly more attractive to J2, but *etr1-1* was not. The lack of phenotype for *etr1-1* is consistent with observations for other ET response phenotypes where it has been attributed to the redundant functions of ET receptors^59^. For example, previous studies reported that an *etr1, etr2*, and *ein4* triple loss-of-function mutant was required for dramatic ET response phenotypes^60^. In addition, the significant attractiveness of plant roots treated with ET-perception inhibitor AgNO_3_ suggests that ET perception by receptors negatively affects the attractiveness of *Arabidopsis* to SCN J2. Taken together, our results indicate that reducing ethylene pathway signaling increases attractiveness of SCN nematode to both host and non-host roots.

The number of nematodes touching the root of the ET-overproducing mutants *eto1-2* and *eto3* was similar to that of WT suggesting that increased ET production of these mutants did not reduce attraction. However, fewer J2 were found in the areas around the root tips of *eto1-2* and *eto3* than of WT, suggesting that increased ET levels or ET signaling in the mutant plants is perceived at a distance from the roots of the non-host plant *Arabidopsis*. However, the constitutive ET-response mutant *ctr1-1* also showed less attraction than WT indicating that a strong, constitutive signaling of ethylene response results in lower attraction. Together these results indicate that strong, constitutive signaling of the ET response results in reduced attraction, but the ET-overproduction of mutants *eto1-2* and *eto3* is not sufficient to be detected in our touching assay, although an effect can be observed at a distance from the root. A possible explanation is that a repellent is produced that diffuses from the root.

Our findings are similar to those obtained using similar assays for the northern root-knot nematode *M. hapla*^35^. The roots of AVG-treated *Arabidopsis* and ET-insensitive mutants displayed increased attraction for *M. hapla.* However, for *M. hapla*, ET-overproducing mutants attracted fewer *M. hapla*, and the roots of GOF-ET receptor mutants *etr1-3* and *ein4-1* both significantly increased the attraction of *M. hapla* compared to WT while our results for SCN found that only *ein4-1* roots were significantly more attractive to SCN J2 than those of the WT. Although *etr1-3* and *ein4-1* identified in genetic screens were all dominant and exhibited partial ET insensitivity^59^,^61^, there was a difference in their insensitivity to ET. Therefore, different attraction between SCN and RKN suggested that RKN are more sensitive than SCN to some consequences of host ethylene signaling.

In contrast to our findings, previous reports found that the roots of ET-overproducing mutant *eto3*^32^ and ETH-treated *Arabidopsis* plants^36^ attracted more J2 of *H. schachtii* than WT, suggesting that ET plays a positive role in the attractiveness of plants to the SBCN *H. schachtii*^32^. The two cyst nematode species, *H. schachtii* and *H. glycines,* are closely related and exhibit the same parasitic behaviors in penetration, migration, and development throughout their life cycle^62^, but differ in host range. There are several possible reasons for the differences regarding the influence of ET signaling between the previous studies and our own work. For example, the designs of the attraction assays differ substantially; we recorded attraction to living root tips after 2 h, whereas Wubben *et al*.^32^, recorded attraction to root-free exudate plugs after 20 h. Thus, our assay reflects a rapid nematode response to an intact root tip, and the other assay may reflect nematode accumulation in response to a host secreted metabolite rather than attraction. It is also possible that these two cyst nematode species recognize or respond to different compounds in the root exudates.

Cyst nematodes cause considerably more wounding during the infection process than do RKN, and the ET response that we see using the ET reporter system may be due to this wounding. Strong *GUS* expression was observed in the infection sites and its surrounding cells up to 24 h after infection indicating an increase in ET signaling at early stages of the *Arabidopsis* - *H. glycines* interaction. In addition, ET is a positive regulator of root hair development, and ET-overproducing mutants^44^,^54^ and ETH-treated *Arabidopsis* plants exhibit the increased number and length of root hairs (Fig. 5a). We also observed root hair proliferation in *EBS::GUS* transgenic seedlings when exposed to SCN infection, further suggesting that ET signaling pathway might be involved in the response of *Arabidopsis* to early SCN infection. In addition, significant increase in the concentration of the ET precursor ACC concentration was found in SCN-infected soybean roots^39^,^63^. These studies suggested that ET pathway plays important roles in host or non-host plant at the early infection and later parasitic stages.

## Materials and Methods

### Nematode culture

A culture of *H. glycines* was initiated with a single cyst isolated from a soybean field in Taian, Shandong Province, China. The single cyst was used to infect the susceptible commercial soybean cultivar Dongsheng1. After 35–40 days, a single cyst collected from the roots was used to initiate the second generation; this process was repeated for 10 generations to produce the strain used for this study. The strain was determined to be SCN race 5 using the SCN differential host test^64^ in a greenhouse in the Northeast Institute of Geography and Agroecology, Harbin, China. Eggs from SCN cysts were incubated in 3 mM ZnSO_4_ solution for hatching at 28 °C, and J2 were collected at 48 h.

### Plant materials and culture

For nematode attraction assays, seeds of soybean cv. DongSheng1 were germinated in pots containing vermiculite in a greenhouse at 22–28 °C with a photoperiod of 16/8 h light/dark cycle, and watered every 3 days with Hoagland’s nutrient solution. The 12-day-old seedling roots were washed with water and lateral roots with intact root tips were used for attraction assays since primary roots were too big to be observed under the microscope.

*Arabidopsis* ecotypes Columbia (Col-0), Wassilewskija (Ws) and Lansbergerecta (Ler), and the ET mutants *ein2-1*^61^, *etr1-3*^61^, and *etr1-7* were provided by Dr. Y. R. Bi (School of Life Sciences, Lanzhou University). The seeds of the *Arabidopsis* transgenic line *EB3::GUS*^57^, mutant lines *ein2-5*^65^, and *eto1-2*^44^ were provided by Dr. H. W. Guo (College of Life Sciences, Peking University). The seeds of mutants *ctr1-1, eto3*^44^, *ein2, ein3-1*^66^, *ein4-1, ein5-1, ein6* (Ler background)^59^, and *ers1*-3 (Ws background)^65^ were obtained from the *Arabidopsis* Biological Resource Center at the Ohio State University. Seeds were surface-sterilized in 15% bleach for 15 min, extensively rinsed with sterilized water, and then placed on plates of half-strength Murashige and Skoog^67^ (1/2 MS) agar medium (pH 5.7) containing 1% (w/v) sucrose and 0.8% (w/v) agar (Biosharp, Japan). After 2–4 days at 4 °C, plates were transferred to a growth chamber where they were kept at an angle of ca. 85° in racks to promote unidirectional root growth. The seedlings were maintained at 22 °C, 16/8 h photoperiod, and photosynthetic photon flux density of 100–120 μM m^‒2^ s^‒1^. After 8 days of growth, *Arabidopsis* roots were cut from seedlings and were used for the attraction assays.

### Nematode attraction

Nematode attraction assays were conducted as previously described^35^,^68^. For soybean assays, 2 ml of 23% (w/v) Pluronic F-127 (NF Prill Poloxamer 407, BASF, Mt Olive, NJ, USA) containing 200 J2s was added into each well of 12-well tissue culture plates at 4 °C; then a 1-cm root piece containing an intact tip was placed into each well. For attraction assays with *Arabidopsis* seedlings, 1 ml 23% PF-127 containing 300 J2 and one root was added into each well of a 12-well tissue culture plate. The plates were transferred to room temperature to allow the gel to solidify.

To optimize the attraction assay, the attractiveness of roots to *H. glycines* was observed microscopically, and the number of J2 touching the root surface up to 5 mm from the root tip was counted at 1 h, 2 h, 3 h, 4 h, and 6 h for soybean or at 1 h, 2 h, 3 h, 4 h, 6 h, and 9 h for *Arabidopsis*. For *Arabidopsis* mutants *ctr1-1, eto1-2*, and *eto3*, the nematodes within the circle area of 5 mm diameter centered at 2.5 mm above the root tips were also counted. Roots and nematodes were photographed with an OLYMPUS SZX-16 dissecting microscope using Cellsens Standard image software (Olympus Corporation, Japan). The experiment was repeated three times with 24 replicates each time.

### Chemical treatments

To evaluate whether ET plays a role in *H. glycines* attraction, 12-day-old soybean plants and 7-day-old *Arabidopsis* plants were treated with the ET analog ethephon (ETH, Sigma-Aldrich), AVG (Sigma-Aldrich) and/or Ag^+^ (Sigma-Aldrich) before starting the attraction assay. The seedling roots were dipped into solutions of 50 μM AVG, and 200 μM ETH and/or 10 μM AgNO_3_. After 24 h, seedling roots of soybean or *Arabidopsis* were washed three times with sterilized water and used for the attraction assays. At 6 h after the attraction assay, ETH and AVG-treated roots were stained with acid fuchsin^69^ and the number of nematodes inside the root was counted. All experiments were repeated at least three times.

### Histochemical analyses

Histochemical staining for GUS activity was performed as described by Jefferson *et al*.^70^ with minor modifications. Seeds of *EB3::GUS* lines were sown on modified Knop medium (pH 6.1) containing 2% (w/v) sucrose, 0.8% (w/v) Daishin agar (Research Products International Corp., USA)^71^. For inoculation, hatching J2s were sterilized in 0.01% mercuric chloride and 0.002% sodium azide for 10 min and immediately washed three times in sterilized water; 50 surface-sterilized J2s were then applied on each twelve-day-old *EB3::GUS* plant. Seedlings were collected at 6, 12, 24 and 48 h after inoculation and then incubated in GUS-staining buffer containing 1 mM X-Gluc, 100 mM sodium phosphate (pH 7.5), 0.5 mM potassium ferrocyanide, 10 mM EDTA and 0.1% Triton X-100 for 12 h at 37 °C in the dark. Individual seedlings were mounted in 50% glycerol on microscopic glass slides and photographed under an Olympus compound microscope using Cellsens Standard image software (Olympus Corporation, Japan). At least 25 replicate plants were analyzed and the experiments were repeated three times.

### Analysis of root hair phenotyping

The number of root hairs was counted in the 500-μm region starting at the primary root tip. The length of root hairs was analyzed by NIH Image software (ImageJ, version 1.43). The average length of root hairs was determined upon measuring 40 hairs for each root. The experiments were repeated at least three times.

### Quantitative reverse transcription PCR (qRT-PCR) analysis

After grinding soybean roots to a fine powder in liquid nitrogen, total RNA was isolated with Trizol (Invitrogen, Carlsbad, CA, USA). RNA samples were digested using RNase-free DNase I (Invitrogen) to eliminate any contaminating genomic DNA. For qRT-PCR analysis, first-strand cDNA was synthesized with 2 μg of total RNA using PrimeScript RT reagent Kit (Thermo Fisher, USA). PCR reactions were performed in the LightCycler^®^ 480 System with FastStart Universal SYBR Green Master (ROX) (Roche) according to the procedure described by the manufacturer. The gene-specific primers were as follows: forward 5′-GGGAAGGGGATGCACACAACCAAGG-3′ and reverse 5′-GTTGGCCATTCCATCCTTCCACCACCT-3′ for *GmERF5*; forward 5′-CAGATTGAGCTTCAGCATTT-3′ and reverse 5′-AAGTGTCATGCTTTGAGGAA-3′ for *GmERS2*; forward 5′-GTGTAATGTTGGATGTGTTCCC-3′ and reverse 5′-ACACAATTGAGTTCAACACAAACCG-3′ for *GmUBQ3*. All PCR cycles began with 10 min at 95 °C, followed by 40 two-step cycles comprising 10 s at 95 °C and 1 min at 60 °C. The relative expression of specific genes was calculated by the 2^−∆∆Ct^ method using *GmUBQ3* as a reference. All experiments were conducted with three independent biological replicates and three technical repetitions.

### Statistical analysis

Data were subjected to one-way analysis of variance (one-way ANOVA) using SPSS software (SAS Institute, Cary, NC, USA). Results are reported as significant or non-significant in Tukey’s *t* Test (Tukey HSD) Test (*P* < *0.05*).

## Additional Information

**How to cite this article**: Hu, Y. *et al*. Ethylene response pathway modulates attractiveness of plant roots to soybean cyst nematode *Heterodera glycines. Sci. Rep.* **7**, 41282; doi: 10.1038/srep41282 (2017).

**Publisher's note:** Springer Nature remains neutral with regard to jurisdictional claims in published maps and institutional affiliations.
